# Investigation of the shared biological mechanisms and common biomarker APTAF1 of sleep deprivation and mild cognitive impairment using integrated bioinformatics analysis

**DOI:** 10.3389/fphar.2024.1387569

**Published:** 2024-04-17

**Authors:** Xiaolan Liu, Baili Lu, Hui Huang

**Affiliations:** ^1^ Wuhan Mental Health Center, Wuhan, Hubei, China; ^2^ Wuhan Hospital for Psychotherapy, Wuhan, Hubei, China

**Keywords:** sleep deprivation, cognitive impairment, mitochondria, machine-learning, biomarkers, immune infiltration

## Abstract

**Introduction:** The relationship between sleep loss and cognitive impairment has long been widely recognized, but there is still a lack of complete understanding of the underlying mechanisms and potential biomarkers. The purpose of this study is to further explore the shared biological mechanisms and common biomarkers between sleep loss and cognitive impairment.

**Methods:** The mitochondria-related genes and gene expression data were downloaded from the MitoCarta3.0 and Gene Expression Omnibus (GEO) databases. We identified the differentially expressed mitochondrial-related genes by combing the differentially expressed genes (DEGs) in sleep deprivation (SD) and mild cognitive impairment (MCI) datasets with mitochondria-related gene lists. Shared DEGs were then further analyzed for enrichment analysis. Next, the common biomarker was identified using two machine learning techniques and further validated using two independent GEO datasets. Then GSEA and GSVA were conducted to analyze the functional categories and pathways enriched for the common biomarker. Finally, immune infiltration analysis was used to investigate the correlation of immune cell infiltration with the common biomarker in SD and MCI.

**Results:** A total of 32 mitochondrial-related differentially expressed genes were identified in SD and MCI. GO analysis indicated that these genes were significantly enriched for mitochondrial transport, and KEGG analysis showed they were mainly involved in pathways of neurodegenerative diseases. In addition, ATPAF1, which was significantly down-regulated in both SD and MCI, was identified through machine learning algorithms as the common biomarker with favorable diagnostic performance. GSEA and GSVA revealed that ATPAF1 was mainly involved in metabolic pathways, such as oxidative phosphorylation, acetylcholine metabolic process, valine, leucine and isoleucine degradation. Immune infiltration analysis showed that the expression of ATPAF1 was correlated with changes in immune cells, especially those key immune cell types associated with SD and MCI.

**Discussion:** This study firstly revealed that mitochondrial dysfunction may be the common pathogenesis of sleep loss and mild cognitive impairment and identified ATPAF1 as a possible biomarker and therapeutic target involved in SD and MCI.

## 1 Introduction

Sleep is a fundamental requirement for both physical and mental health. Unfortunately, sleep problems such as sleep loss are becoming increasingly common in our hectic modern society due to lifestyle changes (Altevogt and Colten, 2006; Grandner, 2012). Despite many unanswered questions regarding the physiological function of sleep and the effects of sleep loss, substantial evidence derived from experimental and clinical studies indicates that sleep loss is associated with various health problems, such as obesity, diabetes (Knutson and Van Cauter, 2008), cardiovascular disease (Tobaldini et al., 2017), and cancer (Chen et al., 2018). In addition, it is generally accepted that getting enough sleep promotes neuronal plasticity, maintains brain health, and enhances cognition; on the other hand, insufficient sleep has the opposite impact and impairs cognitive abilities (Ellenbogen, 2005; Walker and Stickgold, 2006). Moreover, there is increasing epidemiological evidence of a close reciprocal association between mild cognitive impairment (MCI) or Alzheimer’s disease (AD) and sleep problems (Yaffe et al., 2014). For example, a recent meta-analysis revealed that individuals with sleep problems were 1.55-, 1.65-, and 3.78-times more likely to develop AD, MCI, and preclinical AD, respectively, than individuals without sleep problems (Bubu et al., 2017). Therefore, sleep quality is a potentially modifiable risk factor for cognitive decline and AD. Recent studies on acute and chronic SD have elucidated the cellular and molecular mechanisms underlying sleep loss and cognitive deficits. Sleep loss may contribute to cognitive impairment not only by influencing the homeostasis of proteins, such as amyloid-β and tau (Xie et al., 2020; Morrone et al., 2023), but also by modifying neuro-immune cross-talk (Kaneshwaran et al., 2019; Parhizkar et al., 2023). However, a complete understanding of the underlying mechanism is lacking.

Sleep is a period of low metabolic demand that serves metabolic functions (Morselli et al., 2012; Aalling et al., 2018). Given the central metabolic function of mitochondria, they play important roles in regulating sleep and *vice versa* (Hartmann and Kempf, 2023). Besides the production of ATP, mitochondria also perform a variety of functions, ranging from the generation of metabolites and reactive oxygen species (ROS) to the regulation of nuclear gene expression and epigenetics (Kowaltowski et al., 2009; Chandel, 2014; Tatar and Sedivy, 2016). Emerging evidence now indicates that mitochondria are central regulators of cognitive function (Khacho et al., 2019; Alexander et al., 2021). Mitochondrial dysfunction, including impaired mitochondrial biogenesis, disruption of the mitochondrial electron transport chain, increased ROS production, and changes in mitochondrial dynamics, have been observed in both AD and MCI (Gan et al., 2014; Onyango et al., 2016; Apaijai et al., 2020; Wang et al., 2020; Ashleigh et al., 2023). The mechanisms underlying mitochondrial dysfunction induced by sleep problems that trigger cognitive impairment are not well understood.

Nevertheless, there is lack of research combining the transcriptomic signatures of insufficient sleep status in humans with MCI patients for analysis, which may provide an opportunity to investigate the mechanisms of cognitive dysfunction during sleep loss and identify potential biomarkers and treatment strategies. Therefore, this study aimed to investigate the association between SD and MCI using data from the Gene Expression Omnibus (GEO) database. We used differentially expressed genes (DEGs) for functional enrichment analysis and different machine learning approaches for biomarker identification and investigated the diagnostic value of biomarker expression in SD and MCI. In addition, the biological role of this biomarker was analyzed to explore its potential role in disease development and progression. Finally, we determined the proportion of immune cell infiltration related to the biomarker. Using this data, we aimed to provide new insights into the biological mechanisms of SD-induced cognitive impairment and propose new ideas for dual-purpose prevention strategies.

## 2 Materials and methods

### 2.1 Data collection and preprocessing

The GEO database (https://www.ncbi.nlm.nih.gov/geo/) provided all of the gene expression datasets used in this study. Four datasets, numbered GSE39445, GSE140829, GSE56931, and GSE63061, were selected for this study. These datasets were profiled using microarrays of peripheral blood samples. Among them, GSE39445 and GSE56931 showed insufficient sleep status in humans. GSE39445 consists of two groups: individuals underwent SD for 39–41 h as the sleep restriction group, and individuals underwent 10 h of sleep opportunities each day for 1 week as the sleep extension group (Moller-Levet et al., 2013). In GSE56931, participants underwent a 24-h normal sleep/wake cycle (baseline) and 38 h of continuous wakefulness (sleep deprivation), followed by subsequent recovery sleep (recovery) (Arnardottir et al., 2014). In this study, the sleep restriction group in GSE39445 and sleep deprivation group in GSE56931 were considered the SD groups, while the sleep extension group in GSE39445, and baseline and recovery groups in GSE56931 were considered the control groups in our analysis. In addition, GSE140829 and GSE63061 were downloaded from the NCBI GEO database, both of which included gene expression data for patients with MCI and healthy controls. The GSE140829 dataset contained 134 MCI samples and 249 normal samples (Nachun et al., 2019), and GSE63061 contained 110 patients with MCI and 134 control individuals (Sood et al., 2015). In this work, hub genes associated with SD and MCI were found using GSE39445 and GSE140829, and validated using GSE56931 and GSE63061, respectively. For background correction and data normalization, we used the R package “limma” to handle the raw data from these datasets.

### 2.2 Screening mitochondria-related differentially expressed genes

The coding genes for all proteins found in the mitochondrial membrane, matrix, cristae, and mitochondria-associated endoplasmic reticulum membranes were referred to in this study as mitochondria-related genes (MRGs). A total of 1,136 MRGs was acquired from the human MitoCarta3.0 database (https://www.broadinstitute.org/mitocarta/mitocarta30-inventory-mammalian-mitochondrialproteins-and-pathways) (Rath et al., 2021). Next, using the R package “limma” with *p < 0.05*, we carried out a *t*-test difference analysis to find DEGs between SD or MCI and control samples. A heatmap is used to display the resulting differential gene expression data. The intersection of the genes among the MRGs, SD DEGs, and MCI DEGs groups was then shown using the R package “VennDiagram”.

### 2.3 Gene ontology and kyoto encyclopedia of genes and genomes enrichment analysis

The biological roles of DEGs were further investigated by using Kyoto Encyclopedia of Genes and Genomes (KEGG) and Gene Ontology (GO) enrichment analyses. GO analysis is a popular bioinformatics technique for gene annotation of cellular component (CC), molecular function (MF), and biological process (BP), while KEGG is extensively utilized to comprehend biological mechanisms and functions. The GOplot program package was used to visualize the GO and KEGG analyses, with a cutoff *p < 0.05*. Enrichment analysis of target genes in transcription factor targets was performed using the Metascape database (https://metascape.org/gp/index.html#/main/step1).

### 2.4 Identification of key candidate genes using machine learning

The key candidate genes for SD and MCI were screened using two machine learning methods: random forest (RF) and least absolute shrinkage and selection operator (LASSO). LASSO is a regularization and variable selection method for statistical models that constructs a penalty function to solve complex collinear data and develop a relatively refined model (Tibshirani, 2011). RF, on the other hand, is developed by Breiman, which uses decision trees to train classification samples and generates predictions based on the classification outcomes (Breiman, 2001). The R packages “glmnet” (Friedman et al., 2010) and “randomForest” (Breiman, 2018) were used to conduct the LASSO regression and RF analysis.

### 2.5 Differential expression and ROC analysis of the common biomarker

The “limma” R package was utilized to compare the differential expression of the characteristic gene between SD or MCI and control samples in training and validation sets. A boxplot was used to show differences in gene expression. In addition, the diagnostic impact of the characteristic gene in training and validation sets was assessed by computing the area under the curve (AUC) of a receiver-operating characteristic (ROC) curve using the “pROC” R package (Robin et al., 2011).

### 2.6 GSEA and GSVA

The functional categories and pathways enriched for the common biomarker were analyzed using GO and KEGG enrichment analysis based on gene set variation analysis (GSVA) and gene set enrichment analysis (GSEA), in order to further investigate the possible role of the common biomarker in SD and MCI. GSEA and GSVA were carried out using the R packages “clusterprofiler” (Yu et al., 2012) and “GSVA” (Hanzelmann et al., 2013). The reference gene set was determined to be h.all.v6.2.symbols.gmt from the Molecular Signatures Database, using a cut-off criterion of *< 0.05* as a significant threshold.

### 2.7 Immune cell infiltration analysis

The percentage of immune cells in each group was ascertained using the single-sample GSEA (ssGSEA) method. Based on the expression levels of immune cell-related genes generated from the expression profiles, the ssGSEA algorithm was used to examine the infiltration abundance of 28 immune cell types and the correlation between common biomarkers and immune-infiltrating cells. In addition, the packages “limma,” “reshape2,” “tidyverse,” and “ggplot2” were used to evaluate and display the outcomes.

### 2.8 Statistical analysis

Depending on the features of the data distribution, non-parametric tests or t-tests were used to assess the statistical significance of the differences between the two groups. R 4.2.3 was used for all analyses. A threshold of *p < 0.05* was established for statistical significance.

## 3 Result

### 3.1 Screening mitochondria-related differentially expressed genes

**FIGURE 1:**
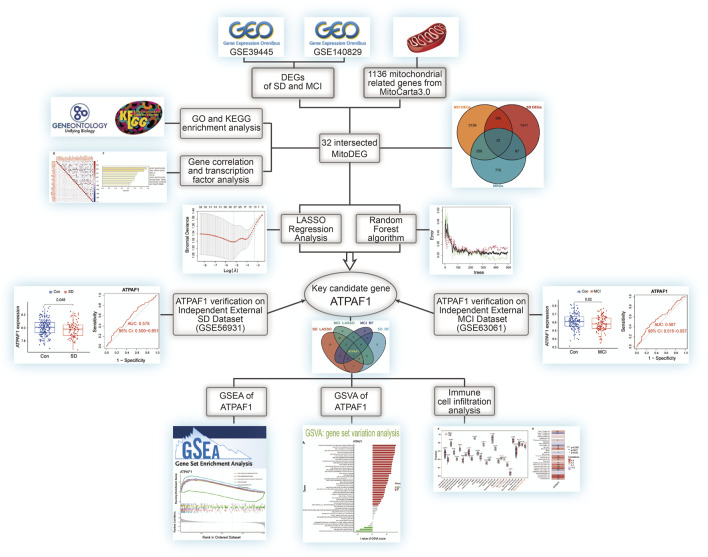
The flowchart of the overall study procedures.

**FIGURE 2:**
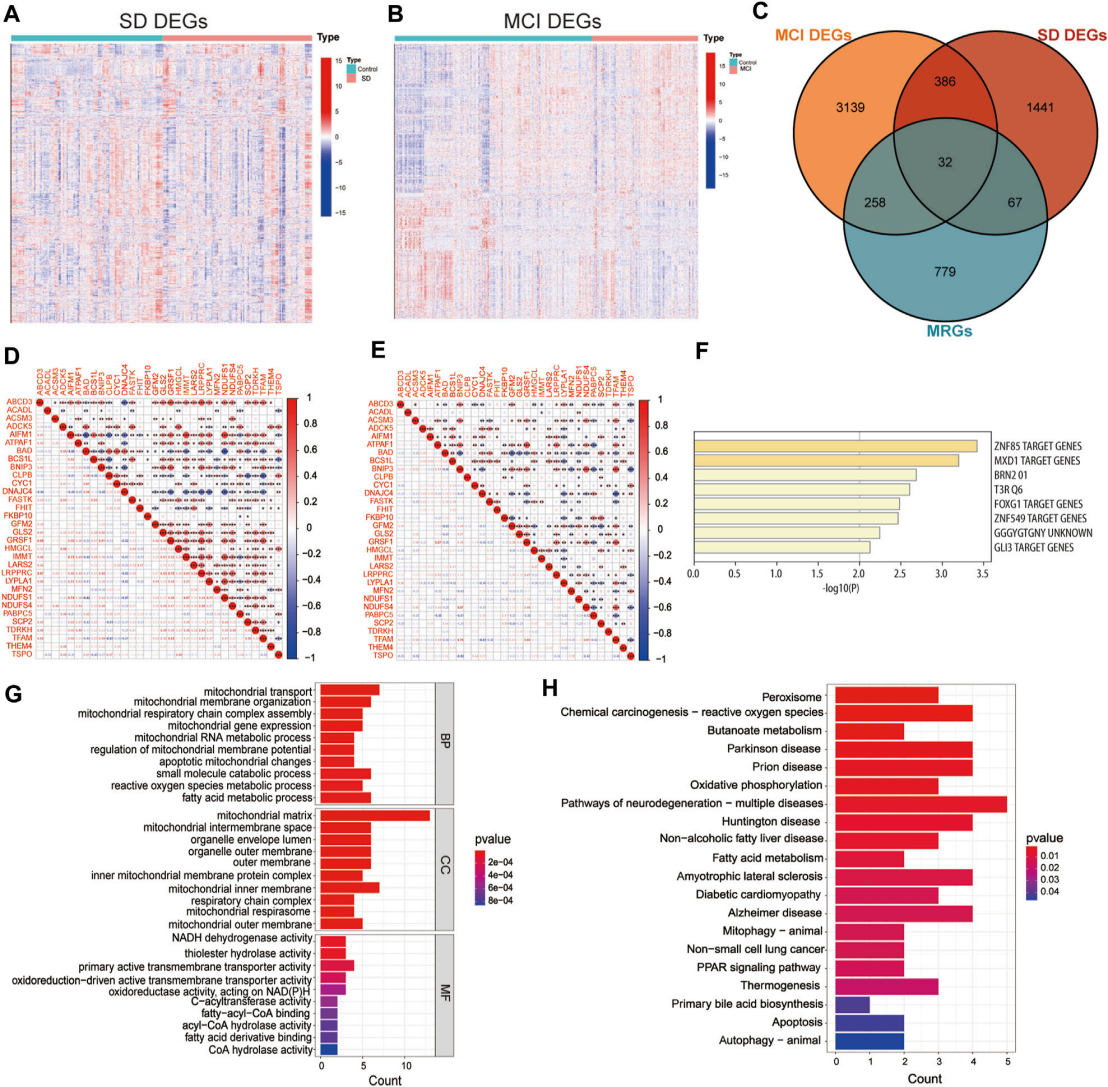
Screening mitochondria-related differentially expressed genes and enrichment analysis **(A)** Heatmap of differentially expressed genes (DEGs) between SD and control group in GSE 39445. The DEGs with upregulated expression are indicated in red, and the DEGs with downregulated expression are shown in blue. **(B)** Heatmap of DEGs between MCI and control group in GSE 140829. The DEGs with upregulated expression are indicated in red, and the DEGs with downregulated expression are shown in blue. **(C)** The Venn diagram shows the overlap of candidate genes among mitochondria-related genes (MRGs), SD DEGs, and MCI DEGs. **(D)** Correlation matrix of the expression levels of the 32 MitoDEGs in GSE 39445. The size of the colored squares represents the strength of the correlation; the red color indicates a positive correlation, and the blue color indicates a negative correlation. * indicates *p* < 0.05, ** indicates *p* < 0.01, *** indicates *p* < 0.001. **(E)** Correlation matrix of the expression levels of the 32 MitoDEGs in GSE 140829. **(F)** The enrichment analysis in Transcription Factor Targets of mitochondrial related differentially expressed genes. **(G)** GO functional enrichment analysis. **(H)** KEGG pathway enrichment analysis.

The flow diagram in Figure 1 served as the guide for conducting this study. We used the expression profiling datasets GSE39445 and GSE140829 to screen for DEGs in the SD and MCI groups, respectively. A total of 1926 DEGs was obtained from the SD dataset, including 561 upregulated DEGs and 1,365 downregulated genes. In addition, 3,815 DEGs were identified in the MCI dataset using differential expression analysis, of which 2,151 genes had upregulated expression, and 1,664 genes had downregulated expression. As shown in Figures 2A, B, we employed heatmaps to present the results of DEGs among different samples in the SD and MCI datasets.

To examine the interrelationship between mitochondrial dysfunction and cognitive impairment induced by SD, we employed the MitoCarta 3.0 database to identify MRGs, and then, performed a cross-comparative analysis to obtain mitochondria-related DEGs. The Venn diagram shows that 32 overlapping MRGs were identified in the SD and MCI datasets (Figure 2C). The majority of the mitochondria-related DEGs were found to be correlated with each other and to exhibit a high and substantial degree of correlation in the SD and MCI datasets, according to our correlation analysis conducted to examine the correlation of gene expression between each of the DEGs (Figures 2D, E).

### 3.2 Gene ontology and kyoto encyclopedia of genes and genomes enrichment analysis

Furthermore, using enrichment analysis of these overlapping genes in transcription factor targets, we identified that the targets of the 32 MitoDEGs were primarily regulated by the zinc finger transcription factor ZNF85 (GO: M30399), transcriptional repressor MXD1 (GO: M40757), and master neural transcription factor BRN2 (GO: M12934), as shown in Figure 2F. Subsequently, we performed GO functional annotation and KEGG pathway enrichment analyses to further understand the biological functions and signaling pathways involved in these DEGs. Figure 2G showed the top 10 GO terms for BP, CC, and MF, respectively. GO analysis showed that mitochondrial transport (GO:0006839) was the most significant biological process, whereas NADH dehydrogenase activity (GO:0003954) and thiolester hydrolase activity (GO:0016790) were the most significant molecular functions. In terms of CC, these genes were mainly distributed in the mitochondrial matrix (GO:0005759).

As shown in Figure 2H, KEGG pathway analysis results revealed the 32 MitoDEGs were mainly enriched in pathways of neurodegenerative diseases, for example, Parkinson’s disease (hsa05012), prion disease (hsa05020), pathways of neurodegeneration-multiple diseases (hsa05022), Huntington’s disease (hsa05016), amyotrophic lateral sclerosis (hsa05014) and Alzheimer disease (hsa05010). The identification of these DEGs could help us obtain a key entry point for studying the cognitive impairment induced by SD, which could lead to an improved understanding of the underlying mechanisms of SD-induced cognitive impairment and facilitate the identification of new therapeutic targets.

### 3.3 Identification of key candidate genes using machine learning

**FIGURE 3:**
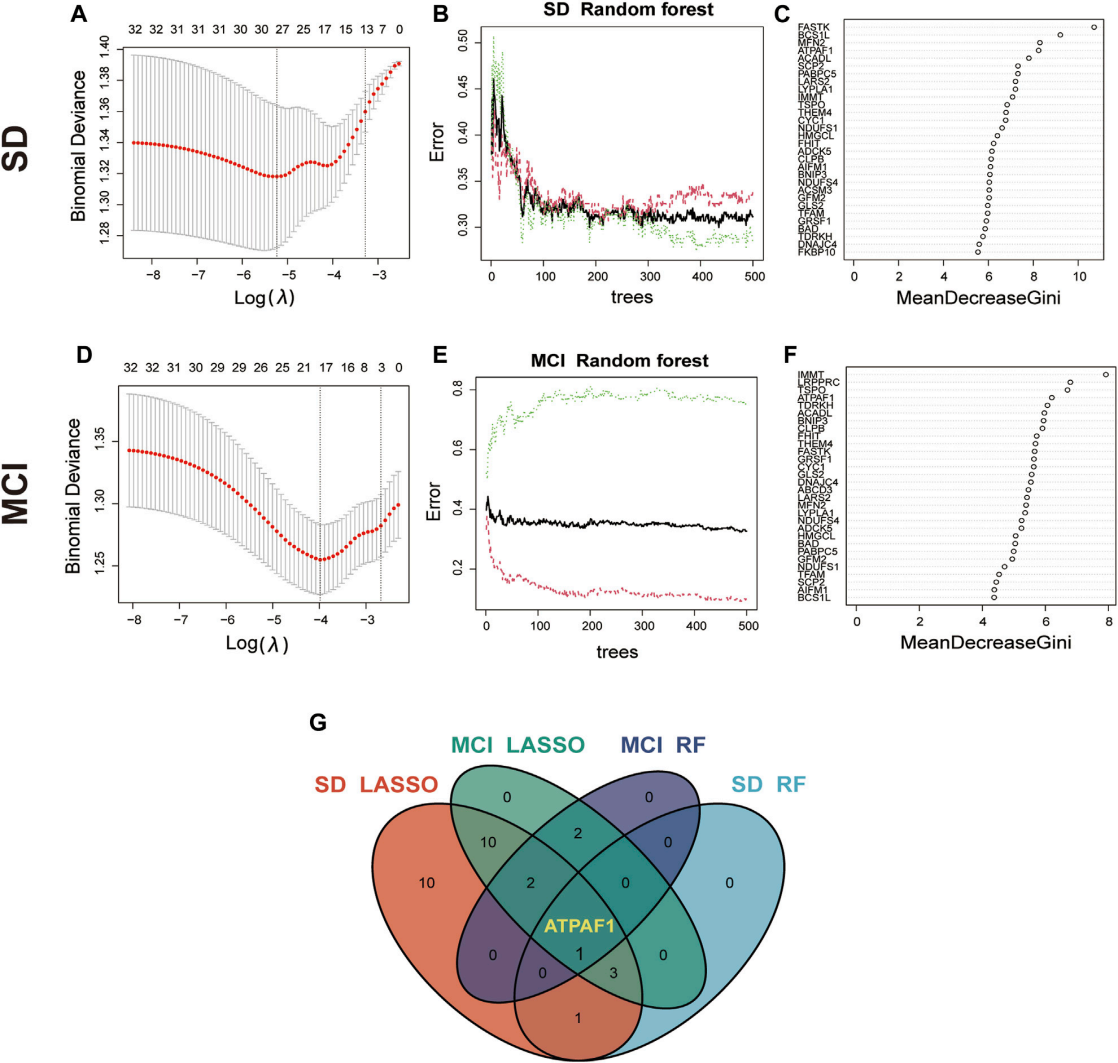
Identification of key candidate genes using machine learning algorithms **(A)** LASSO coefficient profiles of the 32 MitoDEGs in GSE 39445. Cross-validation is used to select the optimal tuning parameter (λ). **(B,C)** Screening candidate genes in GSE 39445 using random forest. The correlation plot between the number of random forest trees and model error is shown. Ranking of input variables in the random forest model based on MeanDecreaseGini to classify SD and control groups. **(D)** LASSO coefficient profiles of the 32 MitoDEGs in GSE 140829. **(E,F)** Screening candidate genes in GSE 140829 using random forest. The correlation plot between the number of random forest trees and model error is shown. Ranking of input variables in the random forest model based on MeanDecreaseGini to classify MCI and control groups. **(G)** The Venn diagram shows the overlap of candidate genes between the four groups.

Based on the aforementioned analysis, it was found that these mitochondria-related DEGs may affect the disease process of cognitive impairment induced by SD through different functions and pathways. Therefore, based on the 32 MitoDEGs, we combined two different machine learning algorithms, LASSO and RF, to screen key DEGs from the SD and MCI datasets. The LASSO logistic regression algorithm identified 27 candidate genes from the 32 MitoDEGs based on the SD dataset GSE39445, and five candidate genes were identified using the RF algorithm (Figures 3A–C). Similarly, based on the MCI dataset GSE140829, 18 candidate genes were identified using LASSO regression, and five candidate genes were identified using the RF algorithm (Figures 3D–F). A venn diagram was plotted to present the overlapping candidate genes among the aforementioned four groups, and ATP synthase mitochondrial F1 complex assembly factor 1 (ATPAF1), a nuclear gene encoding a 31–32-kDa mitochondrial protein essential for ATP synthase F1 assembly and ATP synthase activity, was identified as the key candidate gene (Figure 3G).

### 3.4 Differential expression and ROC analysis of the common biomarker

**FIGURE 4:**
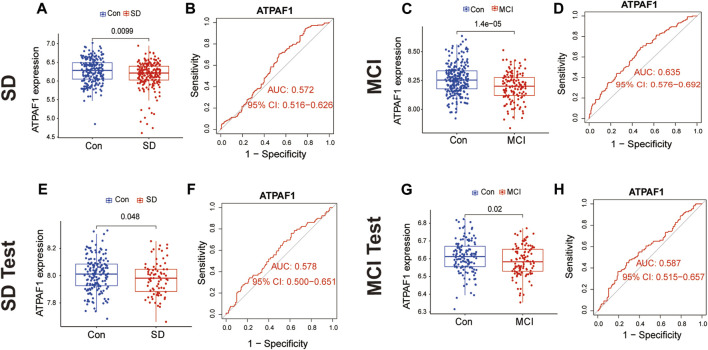
Differential expression and ROC analysis of the common biomarker ATPAF1 **(A,B)** Expression validation and ROC curve analysis of ATPAF1 in the SD training set GSE 39445. Blue indicates the control group, and red indicates the SD group. *p* < 0.05 indicates a significant difference. **(C,D)** Expression validation and ROC curve analysis of ATPAF1 in the MCI training set GSE 140829. Blue indicates the control group, and red indicates the MCI group. *p* < 0.05 indicates a significant difference. **(E,F)** Expression validation and ROC curve analysis of ATPAF1 in the SD validation set GSE 56931. Blue indicates the control group, and red indicates the SD group. *p* < 0.05 indicates a significant difference. **(G,H)** Expression validation and ROC curve analysis of ATPAF1 in the MCI validation set GSE 63061. Blue indicates the control group, and red indicates the MCI group. *p* < 0.05 indicates a significant difference.

Subsequently, we investigated ATPAF1 expression in the SD and MCI datasets. The analysis of expression differences indicated a significant downregulation of ATPAF1 in SD samples in both the training and validation sets (*p < 0.05*; Figures 4A, E). In addition, analysis of the MCI datasets showed that the expression of ATPAF1 was significantly downregulated in both the training and validation sets (Figures 4C, G), suggesting that this gene may play an important role in both cognitive impairment and sleep loss. Moreover, ROC curves were displayed and AUC was computed to distinguish patients with SD and MCI from controls. With AUCs of 0.572 and 0.578 in the SD datasets (Figures 4B, F), and 0.635 and 0.587 in the MCI datasets (Figures 4D, H), ATPAF1 showed a favorable diagnostic value.

### 3.5 GSEA and GSVA

**FIGURE 5:**
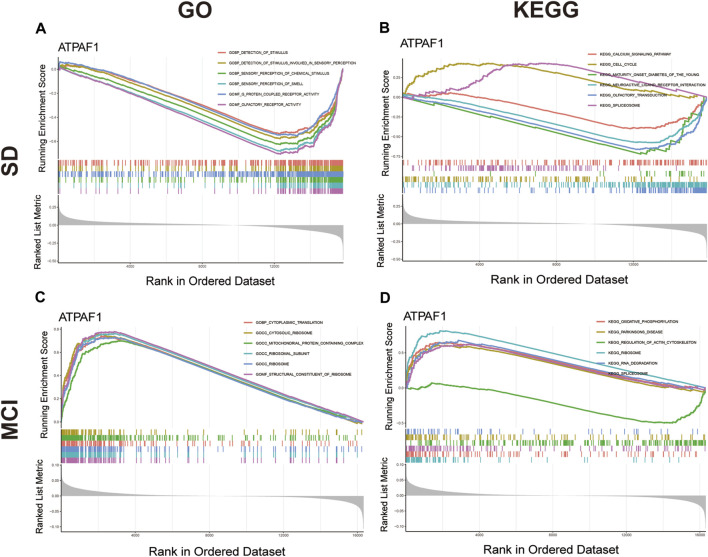
GSEA analysis of ATPAF1 **(A)** GO enrichment analysis of ATPAF1 based on GSEA in GSE 39445. The top six enriched GO terms were shown. **(B)** KEGG enrichment analysis of ATPAF1 based on GSEA in GSE 39445. The top six enriched KEGG pathways were shown. **(C)** GO enrichment analysis of ATPAF1 based on GSEA in GSE 140829. The top six enriched GO terms were shown. **(D)** KEGG enrichment analysis of ATPAF1 based on GSEA in GSE 140829. The top six enriched KEGG pathways were shown.

As ATPAF1 was the key overlapping candidate gene screened by two different machine learning algorithms and its expression was downregulated in both the SD and MCI groups, we speculated that ATPAF1 might be the primary driver among the DEGs and was likely to be an effective target for drug therapy. Therefore, we investigated the biological role of ATPAF1 to explore its potential mechanism in the development of cognitive impairment induced by SD. GSEA was performed to investigate the function and pathways of ATPAF1 in the SD and MCI datasets. According to GSEA analysis results, in the SD dataset, “olfactory receptor activity” and “sensory perception of smell” were the negatively enriched GO terms (Figure 5A), while “olfactory transduction” and “maturity onset diabetes of the young” were the negatively enriched KEGG pathways (Figure 5B). Furthermore, we found that in the MCI dataset, ATPAF1 was positively enriched in “structural constituent of ribosome” and “ribosomal subunit” based on GO terms (Figure 5C), as well as in “ribosome” and “oxidative phosphorylation” based on KEGG pathways (Figure 5D), thus indicating that ATPAF1 is involved in regulating ribosomal protein synthesis and oxidative phosphorylation.

**FIGURE 6:**
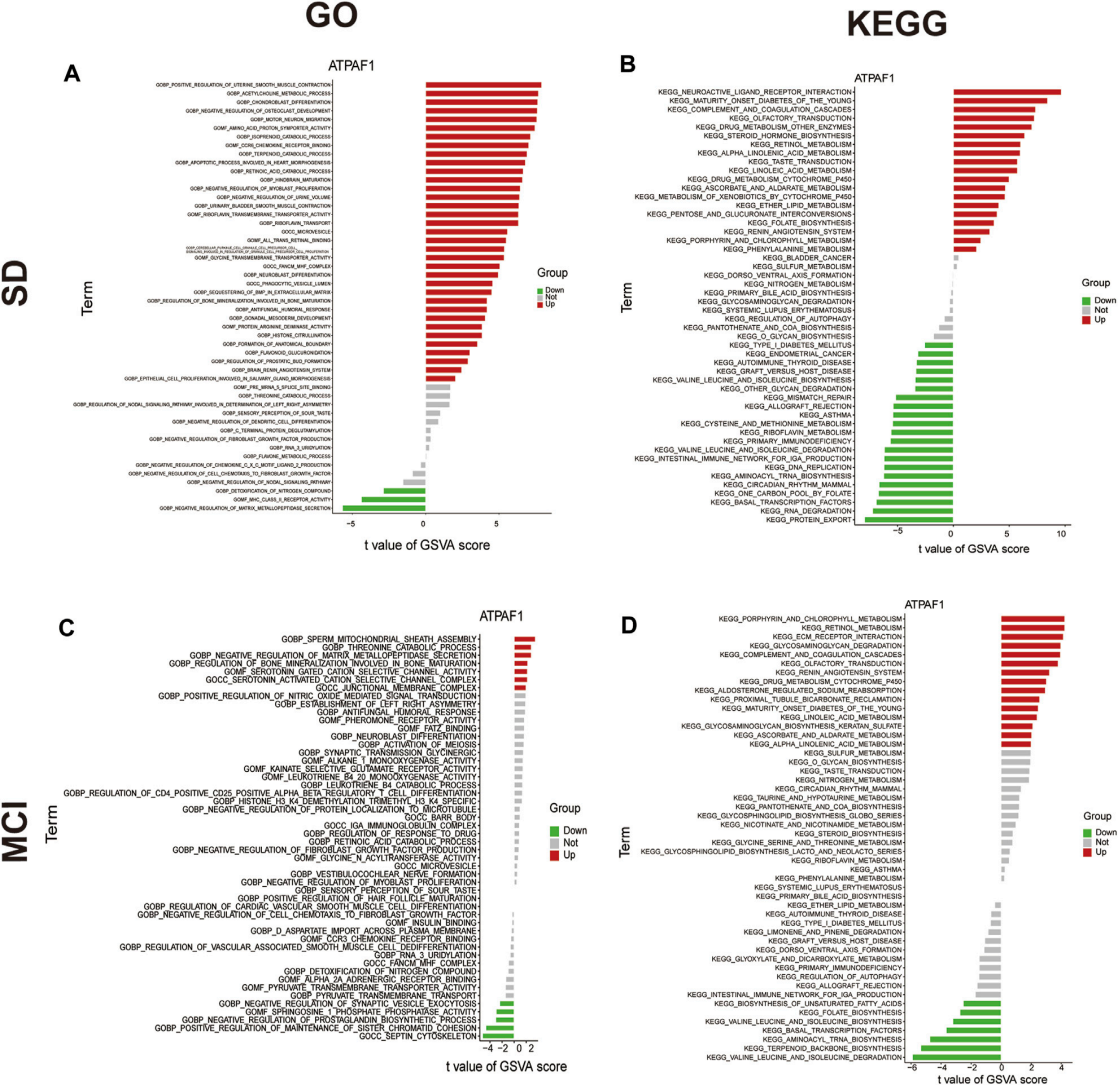
GSVA analysis of ATPAF1 **(A)** GO enrichment analysis of ATPAF1 based on GSVA in GSE 39445. *x*-axis: t value of GSVA score, *y*-axis: GO terms; red indicates upregulated terms, and green indicates downregulated terms. **(B)** KEGG enrichment analysis of ATPAF1 based on GSVA in GSE 39445. *x*-axis: t value of GSVA score, *y*-axis: KEGG pathways; red indicates upregulated pathways, and green indicates downregulated pathways. **(C)** GO enrichment analysis of ATPAF1 based on GSVA in GSE 140829. *x*-axis: t value of GSVA score, *y*-axis: GO terms; red indicates upregulated terms, and green indicates downregulated terms. **(D)** KEGG enrichment analysis of ATPAF1 based on GSVA in GSE 140829. *x*-axis: t value of GSVA score, *y*-axis: KEGG pathways; red indicates upregulated pathways, and green represents downregulated pathways.

Next, GSVA was performed to investigate the possible biological functions of ATPAF1 (Figure 6). Among the enriched GO terms and KEGG pathways, we focused on “acetylcholine metabolic process” in the SD dataset, and “septin cytoskeleton” and “valine leucine and isoleucine degradation” in the MCI dataset. As one of the first neurotransmitters identified in the central nervous system, acetylcholine (ACh) plays a crucial role in learning and memory (Huang et al., 2022). It promotes the conduction of brain nerves and accelerates information transmission, whereas a marked reduction in Ach levels is one of the major characteristics of people with age-related memory loss and AD (Arendt and Bigl, 1986; De Jaeger et al., 2013). Septins, which are enriched in the mammalian nervous system, have been found to be associated with Tau-based paired helical filament core, and contribute to the formation of neurofibrillary tangle in AD (Kinoshita et al., 1998; Hall and Russell, 2004). Furthermore, the effects of the branched-chain amino acids isoleucine, leucine, and valine on cognitive function have been studied previously. Isoleucine, leucine, and valine are significantly associated with the risk of dementia and AD owing to their regulation of the phosphorylation of Tau protein (Tynkkynen et al., 2018).

### 3.6 Immune cell infiltration analysis

**FIGURE 7:**
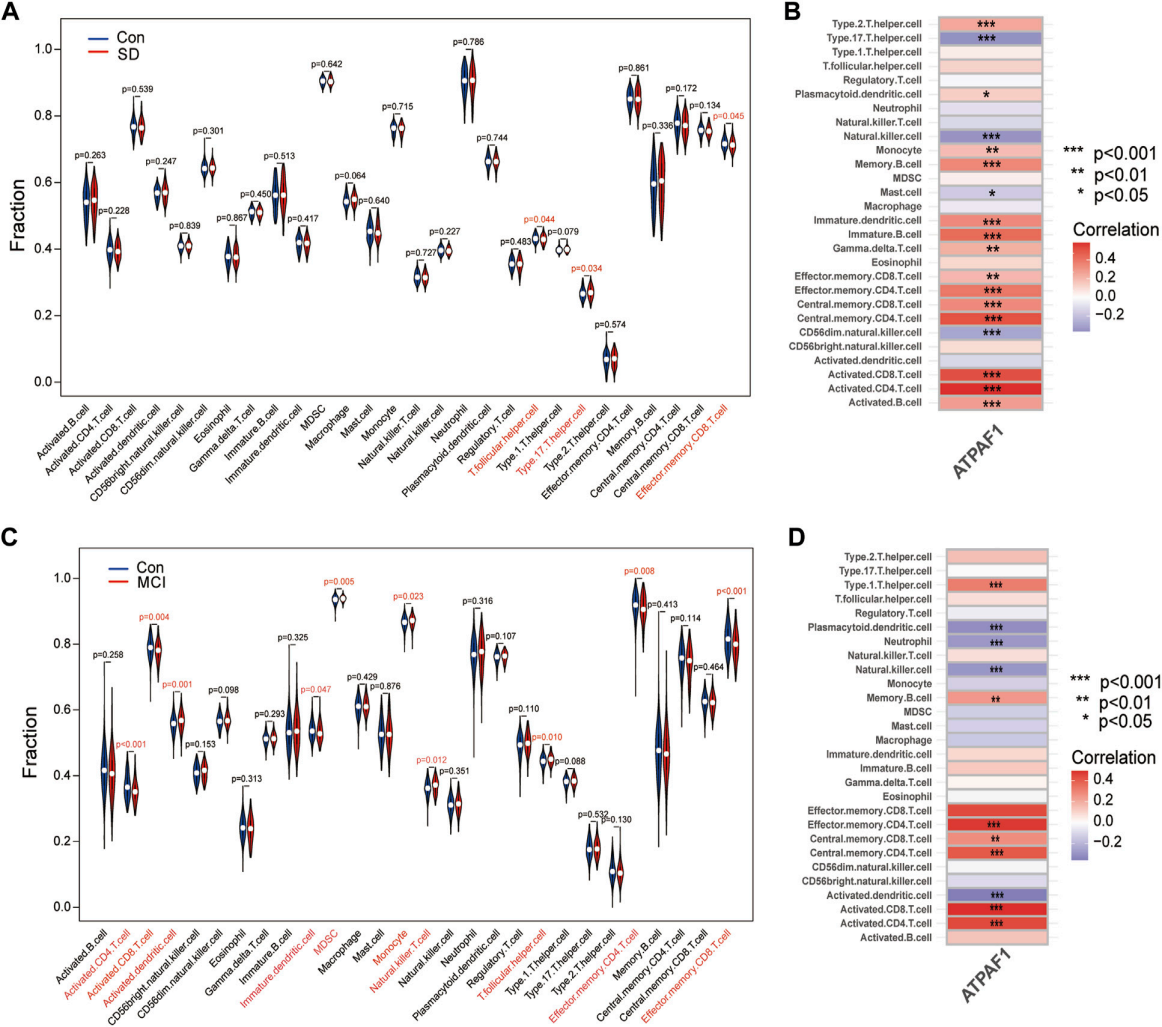
Immune cell infiltration analysis **(A)** The violin plot depicting the difference in immune infiltration between SD and control groups in GSE 39445. Blue indicates the control group, and red indicates the SD group. p < 0.05 indicates a significant difference. **(B)** Correlation analysis of ATPAF1 with the 28 immune infiltrating cells in GSE 39445. * indicates *p* < 0.05, ** indicates *p* < 0.01, *** indicates *p* < 0.001. The redder the color, the higher the positive correlation. The bluer the color, the higher the negative correlation. **(C)** The violin plot depicting the difference in immune infiltration between MCI and control groups in GSE 140829. Blue indicates the control group, and red indicates the MCI group. p < 0.05 indicates a significant difference. **(D)** Correlation analysis of ATPAF1 with the 28 immune infiltrating cells in GSE 140829. * indicates *p < 0.05*, ** indicates *p < 0.01*, *** indicates *p < 0.001*. The redder the color, the higher the positive correlation. The bluer the color, the higher the negative correlation.

Using the ssGSEA method, we examined the percentage of 28 immune cell infiltrations in each sample of the SD dataset in order to examine the differences in immune cell composition between SD and control samples and understand the potential immune mechanisms. The violin plot of immune cell infiltration showed significant differences in T.follicular helper cells, type 17 T helper cells, and effector memory CD8 T cells between the SD and control samples (Figure 7A). Next, the relationship between ATPAF1 expression levels and immune cell abundance was analyzed, which showed that ATPAF1 expression was significantly correlated with 18 types of immune cells, such as type 17 T helper cells and effector memory CD8 T cells (Figure 7B). Similarly, the degree of immune cell infiltration in MCI and control samples was further explored. As shown in Figure 7C, the proportions of activated CD4 T cells, activated CD8 T cells, immature dendritic cells, effector memory CD4 T cells, and effector memory CD8 T cells were significantly lower in the MCI group than in the control, whereas the proportions of activated dendritic cells, MDSC, monocytes, natural killer T cells, and T.follicular helper cells were significantly higher in the MCI group than in the control group. The correlation analysis showed results that ATPAF1 expression was significantly correlated with 11 types of immune cells (Figure 7D). Notably, ATPAF1 was positively correlated with cells such as activated CD4 T cells, activated CD8 T cells, and effector memory CD4 T cells that were significantly lower in number in MCI samples, and negatively correlated with cells such as activated dendritic cells that were significantly higher in number in the MCI group.

## 4 Discussion

Based on a systematic analysis of the transcriptomic signatures of the SD and MCI datasets, this study investigated the shared biological mechanisms that may be involved in sleep loss and cognitive impairment. Our findings helped identify biomarkers of the association between SD and MCI and can be used to explore effective therapeutic interventions in the early stages of disease progression. In this study, 32 overlapping mitochondria-related DEGs between the SD and MCI groups were identified using differential expression profile analysis and cross-comparative analysis. Expression of 10 of the 32 overlapping MitoDEGs was upregulated in both SD and MCI, whereas expression of 13 genes was downregulated in both SD and MCI, as shown in Table 1. Subsequently, GO functional annotation and KEGG pathway enrichment analyses were performed to further understand the biological functions and signaling pathways associated with these DEGs. GO analysis showed that mitochondrial transport was the most significant biological process, and NADH dehydrogenase activity and thiolester hydrolase activity were the most significant molecular functions.

**TABLE 1:** Expression of the 32 overlapping MitoDEGs.

Gene	Description	Expression type in SD	Expression type in MCI	MitoPathways
CYC1	cytochrome c1	Up	Up	OXPHOS > Complex III > CIII subunits | Metabolism > Metals and cofactors > Heme-containing proteins | Metabolism > Electron carriers > Cytochromes | OXPHOS > OXPHOS subunits
LRPPRC	leucine rich pentatricopeptide repeat containing	Down	Down	Mitochondrial central dogma > mtRNA metabolism > mtRNA stability and decay | Mitochondrial central dogma > Translation
NDUFS1	NADH:ubiquinone oxidoreductase core subunit S1	Down	Down	OXPHOS > Complex I > CI subunits | Metabolism > Metals and cofactors > Fe-S-containing proteins | OXPHOS > OXPHOS subunits
LARS2	leucyl-tRNA synthetase 2, mitochondrial	Down	Up	Mitochondrial central dogma > Translation > mt-tRNA synthetases
AIFM1	apoptosis inducing factor mitochondria associated 1	Down	Up	Protein import, sorting and homeostasis > Protein import and sorting > MIA40 | OXPHOS > Complex I > CI assembly factors | OXPHOS > OXPHOS assembly factors | Mitochondrial dynamics and surveillance > Apoptosis
IMMT	inner membrane mitochondrial protein	Down	Up	Mitochondrial dynamics and surveillance > Cristae formation > MICOS complex
ACADL	acyl-CoA dehydrogenase long chain	Down	Down	Metabolism > Lipid metabolism > Fatty acid oxidation
NDUFS4	NADH:ubiquinone oxidoreductase subunit S4	Down	Down	OXPHOS > Complex I > CI subunits | OXPHOS > OXPHOS subunits
GFM2	GTP dependent ribosome recycling factor mitochondrial 2	Down	Down	Mitochondrial central dogma > Translation > Translation factors
BCS1L	BCS1 homolog, ubiquinol-cytochrome c reductase complex chaperone	Up	Up	OXPHOS > Complex III > CIII assembly factors | OXPHOS > OXPHOS assembly factors
CLPB	caseinolytic mitochondrial matrix peptidase chaperone subunit B	Up	Up	Protein import, sorting and homeostasis > Protein homeostasis > Proteases
ADCK5	aarF domain containing kinase 5	Up	Up	unknown
THEM4	thioesterase superfamily member 4	Down	Up	Metabolism > Lipid metabolism
MFN2	mitofusin 2	Down	Up	Mitochondrial dynamics and surveillance > Fusion | Mitochondrial dynamics and surveillance > Organelle contact sites
ACSM3	acyl-CoA synthetase medium chain family member 3	Down	Down	Metabolism > Lipid metabolism > Fatty acid oxidation
HMGCL	3-hydroxy-3-methylglutaryl-CoA lyase	Up	Up	Metabolism > Carbohydrate metabolism > Ketone metabolism | Metabolism > Amino acid metabolism > Branched-chain amino acid metabolism
FHIT	fragile histidine triad diadenosine triphosphatase	Up	Down	Metabolism > Nucleotide metabolism > Nucleotide synthesis and processing
TFAM	transcription factor A, mitochondrial	Down	Down	Mitochondrial central dogma > mtDNA maintainance > mtDNA replication | Mitochondrial central dogma > mtDNA maintainance > mtDNA nucleoid | Mitochondrial central dogma > mtRNA metabolism > Transcription
LYPLA1	lysophospholipase 1	Down	Down	Metabolism > Lipid metabolism | Signaling
ABCD3	ATP binding cassette subfamily D member 3	Down	Down	Small molecule transport > ABC transporters
ATPAF1	ATP synthase mitochondrial F1 complex assembly factor 1	Down	Down	OXPHOS > Complex V > CV assembly factors | OXPHOS > OXPHOS assembly factors
GLS2	glutaminase 2	Down	Down	Metabolism > Amino acid metabolism > Glutamate metabolism
DNAJC4	DnaJ heat shock protein family (Hsp40) member C4	Up	Up	Protein import, sorting and homeostasis > Protein homeostasis
GRSF1	G-rich RNA sequence binding factor 1	Down	Down	Mitochondrial central dogma > mtRNA metabolism > mtRNA granules | Mitochondrial central dogma > mtRNA metabolism > Polycistronic mtRNA processing | Mitochondrial central dogma > mtRNA metabolism > mtRNA stability and decay | Mitochondrial central dogma > Translation > Mitochondrial ribosome assembly
TSPO	translocator protein	Up	Up	Metabolism > Lipid metabolism > Cholesterol, bile acid, steroid synthesis
SCP2	sterol carrier protein 2	Down	Up	Metabolism > Lipid metabolism > Cholesterol, bile acid, steroid synthesis
BNIP3	BCL2 interacting protein 3	Down	Down	Mitochondrial dynamics and surveillance > Apoptosis
FASTK	Fas activated serine/threonine kinase	Up	Up	Mitochondrial central dogma > mtRNA metabolism > mtRNA granules | Mitochondrial central dogma > mtRNA metabolism > Polycistronic mtRNA processing | Mitochondrial central dogma > mtRNA metabolism > mtRNA stability and decay
TDRKH	tudor and KH domain containing	Down	Up	unknown
FKBP10	FKBP prolyl isomerase 10	Down	Up	Protein import, sorting and homeostasis > Protein homeostasis > Chaperones
BAD	BCL2 associated agonist of cell death	Up	Up	Mitochondrial dynamics and surveillance > Apoptosis
PABPC5	poly(A) binding protein cytoplasmic 5	Up	Up	unknown

**FIGURE 8:**
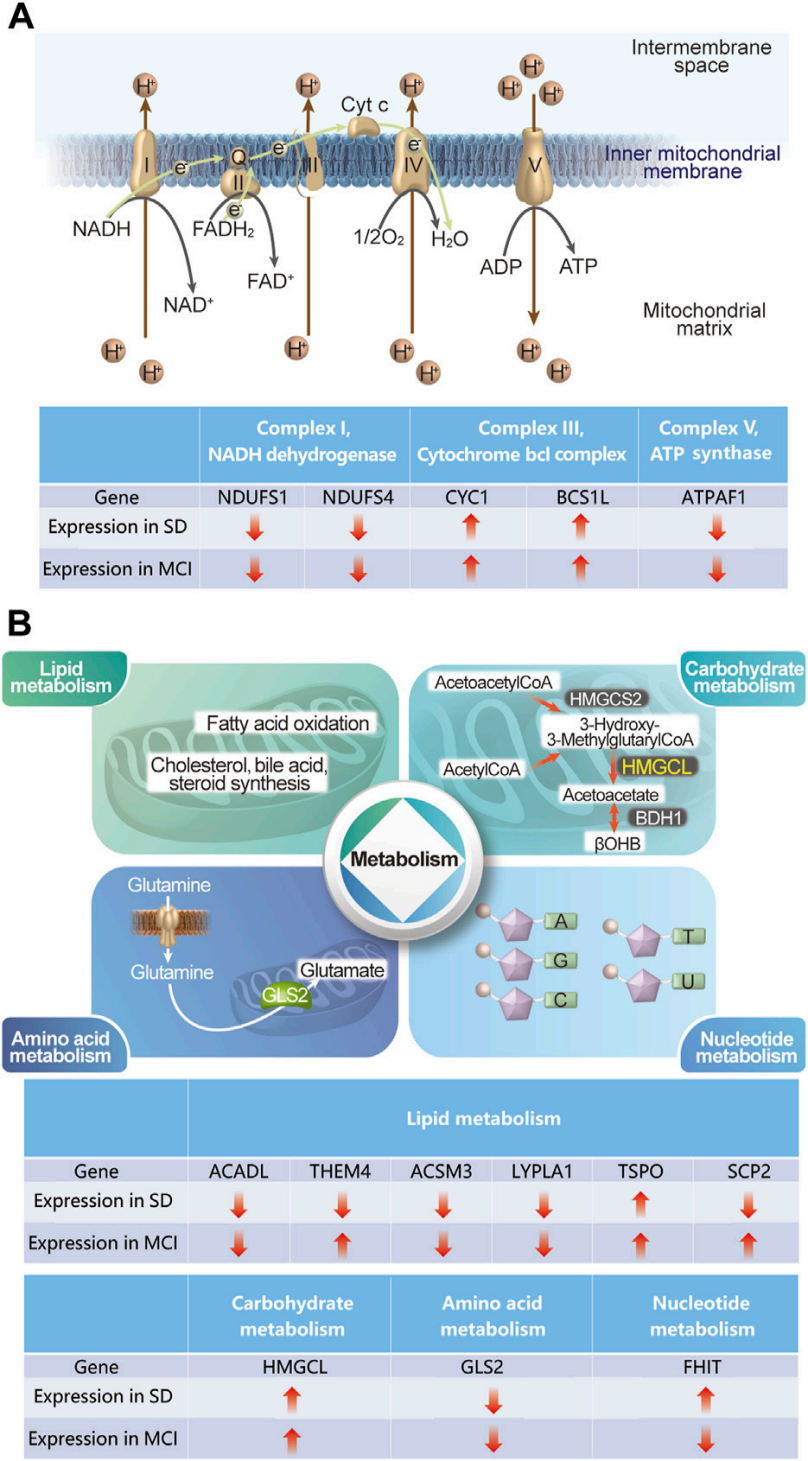
Schematic diagram describing some of the gene expression changes in SD and MCI datasets **(A)** The changes in the five genes in SD and MCI datasets, which encode the components of mitochondria respiratory chain complexes. **(B)** The changes in the nine genes in SD and MCI datasets, which involve lipid metabolism, carbohydrate metabolism, amino acid metabolism, and nucleotide metabolism.

KEGG pathway analysis results revealed that the 32 DEGs were mainly enriched in the pathways of neurodegenerative diseases, such as Parkinson’s disease, prion disease, pathways of neurodegeneration-multiple diseases, Huntington’s disease, amyotrophic lateral sclerosis, and AD. Genes can be annotated to seven major MitoPathways, which are “metabolism,” “central dogma,” “oxidative phosphorylation (OXPHOS),” “mitochondrial dynamics and surveillance,” “protein import, sorting and homeostasis,” “small molecule transport,” and “signaling,” according to the MitoCarta3.0 database. Notably, most of the 32 MitoDEGs were annotated to the MitoPathways of “OXPHOS” and “metabolism.” Among the 32 MitoDEGs, five genes encode components of the mitochondrial respiratory chain complexes, including two from Complex I (NDUFS1 and NDUFS4), two from Complex III (CYC1 and BCS1), and one from Complex V (ATPAF1), as shown in Figure 8A.

Our results regarding the impaired mitochondrial electron transport chain are consistent with those of previous studies on both SD and MCI (Morris et al., 2021; Chen et al., 2022), thus indicating that deficiencies in energy-generating mitochondrial pathways play an important role in cognitive impairment induced by SD. In addition, among the 32 MitoDEGs involved in metabolism, 11 genes are implicated, including six in lipid metabolism (ACADL, THEM4, ACSM3, LYPLA1, TSPO, and SCP2), one in carbohydrate metabolism (HMGCL), one in amino acid metabolism (GLS2), and one in nucleotide metabolism (FHIT), (Figure 8B). Notably, it has been demonstrated that lipid metabolism disorders are closely related to cognitive function, as evidenced by the observation that knockout of a key enzyme in mitochondrial fatty acid oxidation causes cognitive impairment (Zhang et al., 2022; Morant-Ferrando et al., 2023). Importantly, our findings shed light on the role of metabolism, especially lipid metabolism, in the cognitive impairment induced by SD. As mentioned previously, deficiencies in energy-generating mitochondrial pathways and disorders of mitochondrial metabolism link this organelle to the common mechanisms involved in both cognitive impairment and sleep loss.

Based on the 32 MitoDEGs, we employed two machine learning algorithms, LASSO and RF, to screen key candidate genes. ATPAF1 was identified as a key candidate gene through machine learning approaches. As a mitochondria-localized protein, ATPAF1 is essential for ATP synthase assembly and mitochondrial oxidative phosphorylation (Zhou et al., 2021). Although abnormal ATPAF1expression has been reported in asthma and kidney cancer (Schauberger et al., 2011; Bruggemann et al., 2017), its involvement in human diseases remains unclear. In this study, to our knowledge, for the first time, we found that ATPAF1 expression was significantly downregulated in both SD and MCI. Furthermore, the diagnostic ability of ATPAF1 demonstrated favorable value for both diseases. To further explore the potential function of ATPAF1 in SD and MCI, GSVA and GSEA were used to identify functional categories and pathways enriched in ATPAF1. Our results indicate that, by regulating the acetylcholine metabolic process, septin cytoskeleton, and degradation of branched-chain amino acids, ATPAF1 serves as the hub gene shared between SD and MCI. We believe that dual-disease research is a complex and challenging issue, and our findings provide key clues to uncovering the connection between SD and MCI. As the hub gene between SD and MCI, ATPAF1 may link these two diseases through the same or different pathways or functions. Although significant discoveries have been made, the precise mechanisms and interactions of ATPAF1 between SD and MCI have not been fully elucidated in this study, which needs further in-depth investigation.

Sleep is a critical physiological phenomenon involved in immunomodulation at the central and peripheral levels (Hurtado-Alvarado et al., 2016). Several studies have reported an association between sleep loss and systemic low-grade inflammation characterized by the release of several molecules (Aminoff, 2000; Meier-Ewert et al., 2004; Simpson et al., 2017), such as cytokines, chemokines, and acute-phase proteins, which are believed to play important roles in the development of cognitive impairment and AD (Shen et al., 2019; Liao and Yu, 2023). In this study, we performed an immune infiltration analysis to explore the immune landscape of SD and MCI and to further investigate the correlation between ATPAF1 and immune cells. Our results showed that ATPAF1 was positively correlated with cells such as activated CD4 T cells, activated CD8 T cells and effector memory CD4 T cells that were significantly low in number in the MCI samples, and negatively correlated with cells such as activated dendritic cell that were significantly high in number in the MCI group. Similar changes were observed in the SD samples, indicating that ATPAF1 has a statistically significant relationship with immune cells, especially the key immune cell types associated with SD and MCI.

To the best of our knowledge, this is the first study to reveal that mitochondrial dysfunction is a shared biological mechanism underlying sleep loss and cognitive impairment. A total of 32 mitochondria-related DEGs was identified in SD and MCI datasets. Most of them were annotated to the MitoPathways of “OXPHOS” and “metabolism,” thus indicating that deficiencies in energy-generating mitochondrial pathways and disorders of metabolism in mitochondria play an important role in cognitive impairment induced by SD. In addition, ATPAF1 was identified as a possible biomarker and therapeutic target in patients with SD and MCI. This work provides a new perspective on the biological mechanisms of SD-induced cognitive impairment and new ideas for dual-purpose prevention. However, our study was limited to the transcriptome level, and the significance of our findings required further validation through prospective clinical and basic experiments.

## Data Availability

The original contributions presented in the study are included in the article/Supplementary Material, further inquiries can be directed to the corresponding authors.

## Abbreviations

sleep deprivation, SD; mild cognitive impairment, MCI; Gene Expression Omnibus, GEO; Alzheimer’s disease, AD; mitochondria-related genes, MRGs; differentially expressed genes, DEGs; Gene Ontology, GO; Kyoto Encyclopedia of Genes and Genomes, KEGG; biological processes, BP; molecular functions, MF; cellular components, CC; least absolute shrinkage and selection operator, LASSO; random forest, RF; area under the curve, AUC; receiver-operating characteristic, ROC; gene set enrichment analysis, GSEA; gene set variation analysis, GSVA; single-sample gene set enrichment analysis, ssGSEA; ATP synthase mitochondrial F1 complex assembly factor 1, ATPAF1; acetylcholine, ACh; oxidative phosphorylation, OXPHOS.
